# *rac*-4-[4-Cyano-2-(hy­droxy­meth­yl)phen­yl]-4-(4-fluoro­phen­yl)-4-hy­droxy-*N*,*N*-dimethyl­butanaminium hemifumarate

**DOI:** 10.1107/S1600536810054346

**Published:** 2011-01-08

**Authors:** En-Ju Wang, Guang-Ying Chen

**Affiliations:** a Hainan Provincial Key Laboratory of Tropical Pharmaceutical Herb Chemistry, School of Chemistry and Chemical Engineering, Hainan Normal University, Haikou 571158, People’s Republic of China

## Abstract

In the title salt, C_20_H_24_FN_2_O_2_^+^·0.5C_4_H_2_O_4_^2−^, the fumarate anion is located on an inversion centre. In the cation, the two benzene rings are nearly perpendicular to each other, making a dihedral angle of 87.41 (10)°. The cation is linked to the anion by a bifurcated N—H⋯O hydrogen bond. Classical O—H⋯O and weak C—H⋯F hydrogen bonding is also present in the crystal structure. Three C atoms of the *N*,*N*-dimethyl­butanaminium moiety are disordered over two sites with refined site occupancies of 0.466 (14) and 0.534 (14).

## Related literature

For a pharmacological study of the title compound, see: Pollock (2001 ▶). For the synthesis, see: Boegeso (1987 ▶).
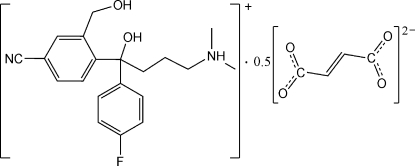


## Experimental

### 

#### Crystal data

C_20_H_24_FN_2_O_2_^+^·0.5C_4_H_2_O_4_^2−^
*M* *_r_* = 400.44
Triclinic, 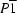

*a* = 8.3312 (9) Å
*b* = 8.8372 (11) Å
*c* = 15.0396 (13) Å
α = 92.236 (1)°
β = 102.681 (2)°
γ = 107.508 (2)°
*V* = 1023.64 (19) Å^3^
*Z* = 2
Mo *K*α radiation
μ = 0.10 mm^−1^
*T* = 293 K
0.50 × 0.48 × 0.47 mm

#### Data collection

Bruker SMART CCD area-detector diffractometer
7766 measured reflections
4381 independent reflections
3071 reflections with *I* > 2σ(*I*)
*R*_int_ = 0.016

#### Refinement

*R*[*F* ^2^ > 2σ(*F* ^2^)] = 0.054
*wR*(*F* ^2^) = 0.158
*S* = 1.06
4381 reflections
297 parameters
H-atom parameters constrained
Δρ_max_ = 0.80 e Å^−3^
Δρ_min_ = −0.29 e Å^−3^

### 

Data collection: *SMART* (Bruker, 2000 ▶); cell refinement: *SAINT* (Bruker, 2000 ▶); data reduction: *SAINT*; program(s) used to solve structure: *SHELXTL* (Sheldrick, 2008 ▶); program(s) used to refine structure: *SHELXTL*; molecular graphics: *SHELXTL*; software used to prepare material for publication: *SHELXTL*.

## Supplementary Material

Crystal structure: contains datablocks I, global. DOI: 10.1107/S1600536810054346/xu5099sup1.cif

Structure factors: contains datablocks I. DOI: 10.1107/S1600536810054346/xu5099Isup2.hkl

Additional supplementary materials:  crystallographic information; 3D view; checkCIF report

## Figures and Tables

**Table 1:** Hydrogen-bond geometry (Å, °)

*D*—H⋯*A*	*D*—H	H⋯*A*	*D*⋯*A*	*D*—H⋯*A*
O1—H1⋯O2	0.82	1.85	2.655 (2)	165
O2—H2⋯O3	0.82	1.85	2.642 (3)	162
N1—H1*A*⋯O4^i^	0.91	2.30	3.152 (3)	155
N1—H1*A*⋯O3^i^	0.91	2.04	2.834 (3)	145
C2—H2*A*⋯F1^ii^	0.93	2.48	3.392 (3)	167

Symmetry codes: (i) 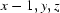; (ii) 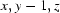.

**Fig. 1.**
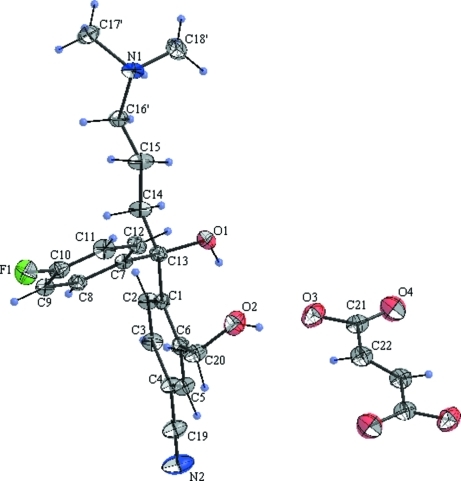
The molecular configuration and atom numbering scheme for (I). Displacement ellipsoids are drawn at the 30% probability level.

**Fig. 2.**
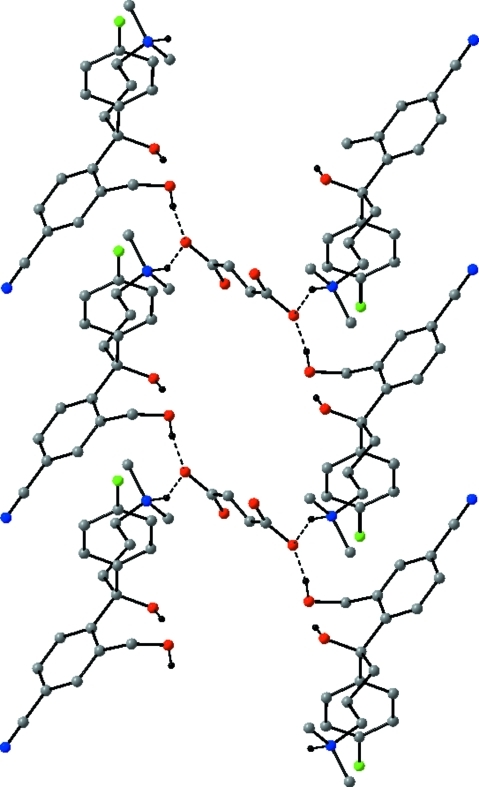
The partial packing diagram of (I), showing a double chain of citalopram diol linked by fumaric acid molecules. Hydrogen bonds are shown as dashed lines. H atoms bonded to C atoms have been omitted for clarity.

**Fig. 3.**
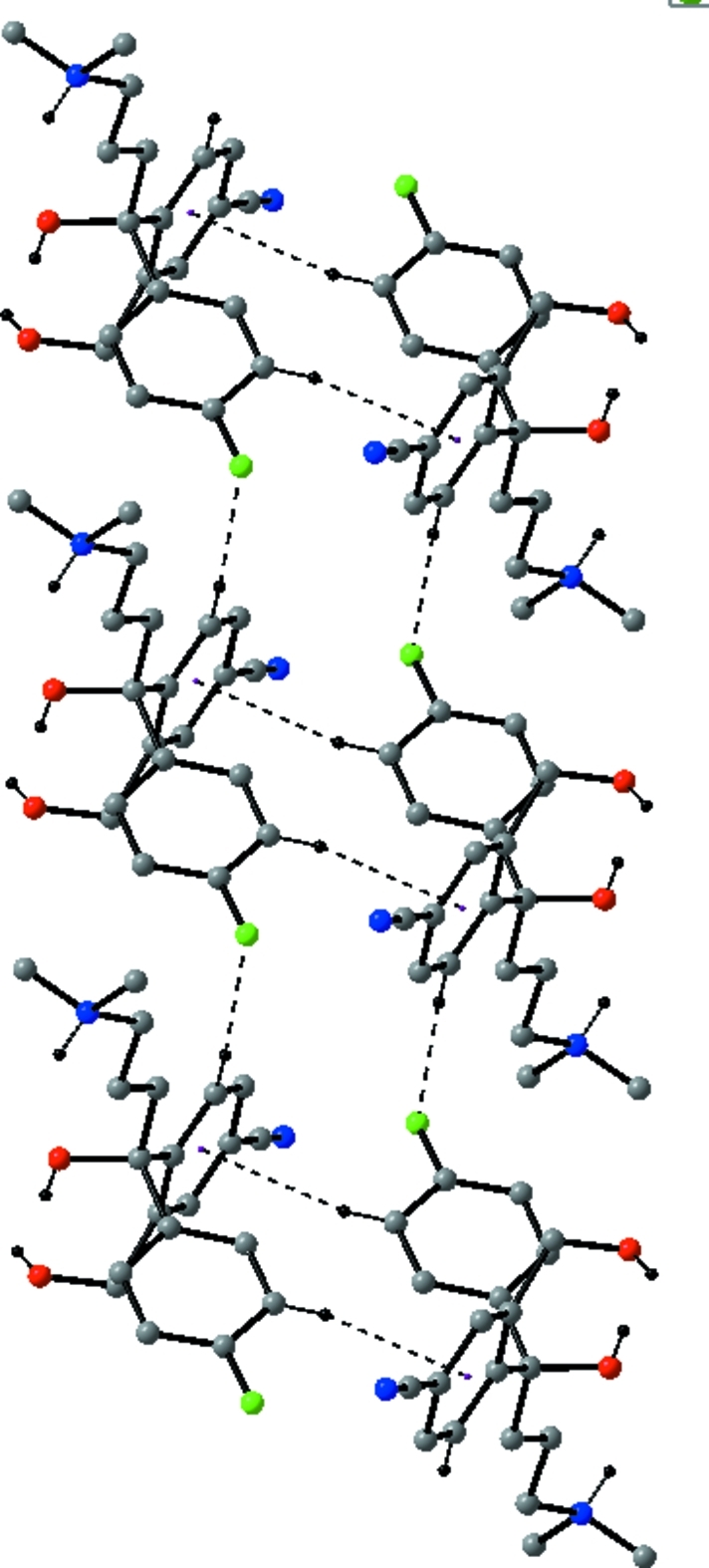
The partial packing diagram of (I), showing the C—H···π and C—H···F interactions. For clarity, some H atoms not involved in the motifs shown have been omitted.

## supplementary crystallographic information

### Comment

*Rac*-4-[4-Cyano-2-(hydroxymethyl)phenyl]-4-(4-fluorophenyl)-4-hydroxy-*N*,*N*-dimethylbutan-1-amine, known as citalopram diol, is a useful intermediate in the synthesis of citalopram that is an efficient antidepressant (Pollock, 2001). Ordinarily both of them are viscous oil and are very difficult to be crystallized. It is a strategy to combine them with all sorts of acid, so that crystal salt can be obtained. The crystal structure of fumaric acid salt of citalopram diol is reported here.

In the title salt, (C_20_H_24_N_2_O_2_F)^ +^.0.5(C_4_H_2_O_4_)^2- ^(I) (Fig. 1), each carboxylic anion of the fumaric acid is involved in two hydrogen bonds and there is an inversion center at the centroid position of fumaric acid, which generate a double chain of citalopram diol linked by fumaric acid molecules (Fig.2). There is a C—H···π interaction between fluorobenzene moiety and benzonitrile moiety (Fig. 3). The dihedral angle between the two aromatic rings is 87.349 (7)°. The distance from H9 to the centroid of aromatic ring of benzonitrile moiety is 2.8372 (8) Å).

### Experimental

The compound was prepared according to the method of patent (Boegeso, 1987). The crystals suitable for single X-ray diffraction were obtained by slowly volatilizing the solution of a mixture of citalopram diol and fumaric acid (2:1 molar ratio) in ethanol.

### Refinement

Hydroxyl H atoms and ammonium H atom were located in a difference Fourier maps and refined with constraints of N—H = 0.91 and O—H = 0.82 Å, *U*_iso_(H) = 1.2*U*_eq_(O,N). Other H atoms were positioned geometrically with C—H = 0.93-0.97 Å, *U*_iso_(H) = 1.5*U*_eq_(C) for methyl and 1.2*U*_eq_(C) for the others. The C16, C17 and C18 atoms were disordered over two sites, occupancies were refined to 0.466 (14):0.534 (14).

### Crystal data

**Table:**

C_20_H_24_FN_2_O_2_^+^·0.5C_4_H_2_O_4_^2^^−^	*Z* = 2
*M**_r_* = 400.44	*F*(000) = 424
Triclinic, *P*1	*D*_x_ = 1.299 Mg m^−^^3^
Hall symbol: -P 1	Mo *K*α radiation, λ = 0.71073 Å
*a* = 8.3312 (9) Å	Cell parameters from 5256 reflections
*b* = 8.8372 (11) Å	θ = 1.5–25.2°
*c* = 15.0396 (13) Å	µ = 0.10 mm^−^^1^
α = 92.236 (1)°	*T* = 293 K
β = 102.681 (2)°	Block, colourless
γ = 107.508 (2)°	0.50 × 0.48 × 0.47 mm
*V* = 1023.64 (19) Å^3^	

### Data collection

**Table:**

Bruker SMART CCD area-detector diffractometer	3071 reflections with *I* > 2σ(*I*)
Radiation source: fine-focus sealed tube	*R*_int_ = 0.016
graphite	θ_max_ = 27.1°, θ_min_ = 1.4°
φ and ω scans	*h* = −10→10
7766 measured reflections	*k* = −11→11
4381 independent reflections	*l* = −19→19

### Refinement

**Table:**

Refinement on *F*^2^	Primary atom site location: structure-invariant direct methods
Least-squares matrix: full	Secondary atom site location: difference Fourier map
*R*[*F*^2^ > 2σ(*F*^2^)] = 0.054	Hydrogen site location: inferred from neighbouring sites
*wR*(*F*^2^) = 0.158	H-atom parameters constrained
*S* = 1.06	*w* = 1/[σ^2^(*F*_o_^2^) + (0.0681*P*)^2^ + 0.4116*P*] where *P* = (*F*_o_^2^ + 2*F*_c_^2^)/3
4381 reflections	(Δ/σ)_max_ = 0.001
297 parameters	Δρ_max_ = 0.80 e Å^−^^3^
0 restraints	Δρ_min_ = −0.29 e Å^−^^3^

### Special details

**Table:**

Geometry. All e.s.d.'s (except the e.s.d. in the dihedral angle between two l.s. planes) are estimated using the full covariance matrix. The cell e.s.d.'s are taken into account individually in the estimation of e.s.d.'s in distances, angles and torsion angles; correlations between e.s.d.'s in cell parameters are only used when they are defined by crystal symmetry. An approximate (isotropic) treatment of cell e.s.d.'s is used for estimating e.s.d.'s involving l.s. planes.
Refinement. Refinement of *F*^2^ against ALL reflections. The weighted *R*-factor *wR* and goodness of fit *S* are based on *F*^2^, conventional *R*-factors *R* are based on *F*, with *F* set to zero for negative *F*^2^. The threshold expression of *F*^2^ > σ(*F*^2^) is used only for calculating *R*-factors(gt) *etc*. and is not relevant to the choice of reflections for refinement. *R*-factors based on *F*^2^ are statistically about twice as large as those based on *F*, and *R*- factors based on ALL data will be even larger.

### Fractional atomic coordinates and isotropic or equivalent isotropic displacement parameters (Å^2^)

**Table:**

	*x*	*y*	*z*	*U*_iso_*/*U*_eq_	Occ. (<1)
F1	0.20769 (19)	0.79749 (15)	0.86453 (10)	0.0671 (4)	
N1	−0.2494 (2)	−0.04111 (19)	0.61890 (12)	0.0480 (4)	
H1A	−0.2346	0.0452	0.5868	0.058*	
N2	1.1468 (3)	0.2082 (3)	1.02194 (19)	0.0865 (8)	
O1	0.38654 (19)	0.25106 (19)	0.66277 (9)	0.0536 (4)	
H1	0.4688	0.3252	0.6562	0.080*	
O2	0.6756 (2)	0.4911 (2)	0.67399 (11)	0.0690 (5)	
H2	0.7266	0.4358	0.6545	0.104*	
O3	0.7715 (2)	0.2781 (2)	0.58894 (13)	0.0765 (5)	
O4	0.8536 (3)	0.1944 (2)	0.47374 (15)	0.0906 (6)	
C1	0.5496 (2)	0.2512 (2)	0.81662 (12)	0.0382 (4)	
C2	0.5520 (3)	0.1242 (2)	0.86801 (13)	0.0450 (4)	
H2A	0.4472	0.0468	0.8671	0.054*	
C3	0.7026 (3)	0.1076 (2)	0.92031 (14)	0.0479 (5)	
H3	0.6992	0.0202	0.9532	0.057*	
C4	0.8584 (3)	0.2230 (3)	0.92298 (14)	0.0482 (5)	
C5	0.8597 (3)	0.3513 (3)	0.87273 (15)	0.0505 (5)	
H5	0.9654	0.4280	0.8745	0.061*	
C6	0.7095 (2)	0.3695 (2)	0.82005 (13)	0.0445 (4)	
C7	0.3417 (2)	0.4110 (2)	0.78555 (12)	0.0374 (4)	
C8	0.3553 (2)	0.4536 (2)	0.87729 (13)	0.0414 (4)	
H8	0.3930	0.3925	0.9212	0.050*	
C9	0.3138 (3)	0.5849 (2)	0.90451 (14)	0.0460 (5)	
H9	0.3241	0.6138	0.9661	0.055*	
C10	0.2568 (3)	0.6718 (2)	0.83815 (15)	0.0470 (5)	
C11	0.2425 (3)	0.6358 (2)	0.74730 (15)	0.0510 (5)	
H11	0.2047	0.6978	0.7040	0.061*	
C12	0.2858 (3)	0.5040 (2)	0.72121 (14)	0.0477 (5)	
H12	0.2773	0.4775	0.6595	0.057*	
C13	0.3780 (2)	0.2592 (2)	0.75605 (12)	0.0405 (4)	
C14	0.2230 (2)	0.1135 (2)	0.75868 (15)	0.0481 (5)	
H14A	0.2482	0.0170	0.7430	0.058*	
H14B	0.2064	0.1117	0.8205	0.058*	
C15	0.0558 (3)	0.1151 (3)	0.69250 (17)	0.0589 (6)	
H15A	0.0034	0.1812	0.7216	0.071*	
H15B	0.0834	0.1619	0.6383	0.071*	
C16	−0.0679 (8)	−0.0449 (7)	0.6654 (7)	0.0476 (17)	0.466 (14)
H16A	−0.0737	−0.1014	0.7192	0.057*	0.466 (14)
H16B	−0.0279	−0.1029	0.6235	0.057*	0.466 (14)
C17	−0.3573 (14)	−0.0310 (15)	0.6699 (7)	0.081 (3)	0.466 (14)
H17A	−0.4580	−0.0138	0.6323	0.121*	0.466 (14)
H17B	−0.3005	0.0565	0.7174	0.121*	0.466 (14)
H17C	−0.3919	−0.1285	0.6971	0.121*	0.466 (14)
C18	−0.3287 (14)	−0.1936 (14)	0.5463 (7)	0.072 (2)	0.466 (14)
H18A	−0.3457	−0.2878	0.5774	0.109*	0.466 (14)
H18B	−0.2506	−0.1931	0.5078	0.109*	0.466 (14)
H18C	−0.4380	−0.1927	0.5093	0.109*	0.466 (14)
C16'	−0.1003 (8)	−0.0176 (9)	0.7010 (5)	0.0522 (17)	0.534 (14)
H16C	−0.1343	0.0073	0.7560	0.063*	0.534 (14)
H16D	−0.0713	−0.1157	0.7065	0.063*	0.534 (14)
C17'	−0.4188 (10)	−0.0984 (12)	0.6542 (6)	0.0696 (19)	0.534 (14)
H17D	−0.5174	−0.1059	0.6051	0.104*	0.534 (14)
H17E	−0.4126	−0.0232	0.7037	0.104*	0.534 (14)
H17F	−0.4304	−0.2012	0.6755	0.104*	0.534 (14)
C18'	−0.2597 (14)	−0.1404 (10)	0.5439 (6)	0.069 (2)	0.534 (14)
H18D	−0.2704	−0.2459	0.5609	0.104*	0.534 (14)
H18E	−0.1566	−0.1009	0.5219	0.104*	0.534 (14)
H18F	−0.3592	−0.1438	0.4964	0.104*	0.534 (14)
C19	1.0189 (3)	0.2131 (3)	0.97840 (17)	0.0611 (6)	
C20	0.7315 (3)	0.5186 (3)	0.77151 (16)	0.0566 (6)	
H20A	0.6673	0.5809	0.7936	0.068*	
H20B	0.8532	0.5823	0.7882	0.068*	
C21	0.8499 (3)	0.3012 (3)	0.52575 (16)	0.0546 (5)	
C22	0.9421 (3)	0.4749 (3)	0.52195 (16)	0.0596 (6)	
H22	0.9121	0.5504	0.5538	0.072*	

### Atomic displacement parameters (Å^2^)

**Table:**

	*U*^11^	*U*^22^	*U*^33^	*U*^12^	*U*^13^	*U*^23^
F1	0.0795 (9)	0.0485 (7)	0.0819 (9)	0.0300 (7)	0.0234 (7)	0.0074 (6)
N1	0.0391 (9)	0.0413 (9)	0.0524 (10)	0.0045 (7)	−0.0009 (7)	0.0088 (7)
N2	0.0497 (13)	0.0901 (17)	0.1124 (19)	0.0268 (12)	−0.0048 (12)	0.0306 (14)
O1	0.0550 (9)	0.0639 (10)	0.0372 (7)	0.0175 (7)	0.0052 (6)	−0.0027 (6)
O2	0.0677 (11)	0.0855 (13)	0.0646 (10)	0.0316 (9)	0.0234 (8)	0.0325 (9)
O3	0.0687 (11)	0.0924 (13)	0.0771 (12)	0.0283 (10)	0.0276 (9)	0.0315 (10)
O4	0.0964 (15)	0.0679 (12)	0.1023 (15)	0.0192 (11)	0.0256 (12)	−0.0067 (11)
C1	0.0357 (9)	0.0409 (10)	0.0369 (9)	0.0136 (8)	0.0053 (7)	−0.0006 (7)
C2	0.0382 (10)	0.0433 (10)	0.0497 (11)	0.0112 (8)	0.0050 (8)	0.0046 (8)
C3	0.0468 (11)	0.0455 (11)	0.0516 (11)	0.0182 (9)	0.0067 (9)	0.0084 (9)
C4	0.0384 (10)	0.0575 (12)	0.0504 (11)	0.0216 (9)	0.0056 (8)	0.0052 (9)
C5	0.0344 (10)	0.0556 (12)	0.0601 (13)	0.0113 (9)	0.0117 (9)	0.0112 (10)
C6	0.0399 (10)	0.0489 (11)	0.0454 (10)	0.0149 (8)	0.0103 (8)	0.0078 (8)
C7	0.0307 (9)	0.0386 (9)	0.0388 (9)	0.0088 (7)	0.0031 (7)	0.0043 (7)
C8	0.0360 (10)	0.0474 (10)	0.0397 (10)	0.0149 (8)	0.0038 (8)	0.0082 (8)
C9	0.0408 (10)	0.0527 (11)	0.0425 (10)	0.0142 (9)	0.0078 (8)	0.0018 (8)
C10	0.0416 (11)	0.0369 (10)	0.0616 (13)	0.0114 (8)	0.0122 (9)	0.0057 (8)
C11	0.0540 (12)	0.0461 (11)	0.0522 (12)	0.0184 (9)	0.0066 (9)	0.0152 (9)
C12	0.0507 (12)	0.0506 (11)	0.0388 (10)	0.0158 (9)	0.0048 (8)	0.0082 (8)
C13	0.0369 (10)	0.0442 (10)	0.0367 (9)	0.0123 (8)	0.0026 (7)	0.0021 (7)
C14	0.0384 (10)	0.0422 (10)	0.0552 (12)	0.0099 (8)	−0.0015 (9)	0.0037 (8)
C15	0.0396 (11)	0.0547 (13)	0.0701 (15)	0.0084 (9)	−0.0033 (10)	0.0108 (10)
C16	0.038 (3)	0.049 (3)	0.052 (4)	0.015 (2)	0.001 (2)	0.001 (2)
C17	0.048 (5)	0.092 (7)	0.101 (6)	0.020 (4)	0.023 (4)	−0.013 (5)
C18	0.058 (5)	0.082 (7)	0.054 (4)	0.004 (4)	−0.005 (4)	−0.011 (4)
C16'	0.039 (3)	0.059 (3)	0.047 (3)	0.006 (2)	0.002 (2)	0.009 (2)
C17'	0.040 (4)	0.073 (5)	0.090 (5)	0.007 (3)	0.019 (3)	0.006 (4)
C18'	0.078 (6)	0.059 (4)	0.059 (3)	0.006 (3)	0.019 (4)	−0.012 (3)
C19	0.0466 (13)	0.0629 (14)	0.0733 (15)	0.0206 (11)	0.0077 (11)	0.0161 (11)
C20	0.0431 (11)	0.0581 (13)	0.0661 (14)	0.0117 (10)	0.0120 (10)	0.0216 (11)
C21	0.0453 (12)	0.0589 (13)	0.0558 (13)	0.0126 (10)	0.0071 (10)	0.0225 (11)
C22	0.0566 (14)	0.0654 (14)	0.0569 (13)	0.0211 (11)	0.0096 (10)	0.0180 (11)

### Geometric parameters (Å, °)

**Table:**

F1—C10	1.367 (2)	C10—C11	1.362 (3)
N1—C17	1.323 (9)	C11—C12	1.386 (3)
N1—C18'	1.373 (8)	C11—H11	0.9300
N1—C16'	1.504 (5)	C12—H12	0.9300
N1—C16	1.531 (6)	C13—C14	1.534 (3)
N1—C17'	1.566 (8)	C14—C15	1.528 (3)
N1—C18	1.574 (10)	C14—H14A	0.9700
N1—H1A	0.9100	C14—H14B	0.9700
N2—C19	1.136 (3)	C15—C16	1.456 (6)
O1—C13	1.420 (2)	C15—C16'	1.500 (6)
O1—H1	0.8200	C15—H15A	0.9700
O2—C20	1.425 (3)	C15—H15B	0.9700
O2—H2	0.8200	C16—H16A	0.9700
O3—C21	1.257 (3)	C16—H16B	0.9700
O4—C21	1.214 (3)	C17—H17A	0.9600
C1—C2	1.391 (3)	C17—H17B	0.9600
C1—C6	1.414 (3)	C17—H17C	0.9600
C1—C13	1.540 (2)	C18—H18A	0.9600
C2—C3	1.378 (3)	C18—H18B	0.9600
C2—H2A	0.9300	C18—H18C	0.9600
C3—C4	1.380 (3)	C16'—H16C	0.9700
C3—H3	0.9300	C16'—H16D	0.9700
C4—C5	1.386 (3)	C17'—H17D	0.9600
C4—C19	1.441 (3)	C17'—H17E	0.9600
C5—C6	1.384 (3)	C17'—H17F	0.9600
C5—H5	0.9300	C18'—H18D	0.9600
C6—C20	1.514 (3)	C18'—H18E	0.9600
C7—C12	1.386 (3)	C18'—H18F	0.9600
C7—C8	1.387 (3)	C20—H20A	0.9700
C7—C13	1.531 (3)	C20—H20B	0.9700
C8—C9	1.379 (3)	C21—C22	1.506 (3)
C8—H8	0.9300	C22—C22^i^	1.269 (4)
C9—C10	1.372 (3)	C22—H22	0.9300
C9—H9	0.9300		
			
C17—N1—C18'	134.8 (5)	O1—C13—C1	109.05 (15)
C17—N1—C16'	93.0 (5)	C7—C13—C1	110.65 (14)
C18'—N1—C16'	117.9 (5)	C14—C13—C1	112.41 (15)
C17—N1—C16	119.4 (5)	C15—C14—C13	112.24 (17)
C18'—N1—C16	92.2 (4)	C15—C14—H14A	109.2
C16'—N1—C16	27.2 (2)	C13—C14—H14A	109.2
C17—N1—C17'	24.3 (4)	C15—C14—H14B	109.2
C18'—N1—C17'	110.8 (4)	C13—C14—H14B	109.2
C16'—N1—C17'	106.5 (4)	H14A—C14—H14B	107.9
C16—N1—C17'	128.0 (5)	C16—C15—C16'	27.9 (2)
C17—N1—C18	111.7 (5)	C16—C15—C14	111.9 (3)
C18'—N1—C18	23.5 (4)	C16'—C15—C14	112.9 (3)
C16'—N1—C18	124.9 (6)	C16—C15—H15A	109.2
C16—N1—C18	104.7 (5)	C16'—C15—H15A	83.4
C17'—N1—C18	87.5 (4)	C14—C15—H15A	109.2
C17—N1—H1A	106.8	C16—C15—H15B	109.2
C18'—N1—H1A	92.0	C16'—C15—H15B	129.4
C16'—N1—H1A	111.9	C14—C15—H15B	109.2
C16—N1—H1A	106.8	H15A—C15—H15B	107.9
C17'—N1—H1A	117.7	C15—C16—N1	111.9 (4)
C18—N1—H1A	106.8	C15—C16—H16A	109.2
C13—O1—H1	109.5	N1—C16—H16A	109.2
C20—O2—H2	109.5	C15—C16—H16B	109.2
C2—C1—C6	117.88 (17)	N1—C16—H16B	109.2
C2—C1—C13	120.62 (16)	H16A—C16—H16B	107.9
C6—C1—C13	121.48 (16)	N1—C17—H17A	109.5
C3—C2—C1	123.02 (18)	N1—C17—H17B	109.5
C3—C2—H2A	118.5	N1—C17—H17C	109.5
C1—C2—H2A	118.5	N1—C18—H18A	109.5
C2—C3—C4	118.84 (19)	N1—C18—H18B	109.5
C2—C3—H3	120.6	N1—C18—H18C	109.5
C4—C3—H3	120.6	C15—C16'—N1	111.1 (4)
C3—C4—C5	119.35 (18)	C15—C16'—H16C	109.4
C3—C4—C19	120.94 (19)	N1—C16'—H16C	109.4
C5—C4—C19	119.7 (2)	C15—C16'—H16D	109.4
C6—C5—C4	122.44 (19)	N1—C16'—H16D	109.4
C6—C5—H5	118.8	H16C—C16'—H16D	108.0
C4—C5—H5	118.8	N1—C17'—H17D	109.5
C5—C6—C1	118.47 (18)	N1—C17'—H17E	109.5
C5—C6—C20	116.23 (18)	H17D—C17'—H17E	109.5
C1—C6—C20	125.30 (18)	N1—C17'—H17F	109.5
C12—C7—C8	118.55 (17)	H17D—C17'—H17F	109.5
C12—C7—C13	121.01 (16)	H17E—C17'—H17F	109.5
C8—C7—C13	120.36 (16)	N1—C18'—H18D	109.5
C9—C8—C7	121.12 (17)	N1—C18'—H18E	109.5
C9—C8—H8	119.4	H18D—C18'—H18E	109.5
C7—C8—H8	119.4	N1—C18'—H18F	109.5
C10—C9—C8	118.15 (18)	H18D—C18'—H18F	109.5
C10—C9—H9	120.9	H18E—C18'—H18F	109.5
C8—C9—H9	120.9	N2—C19—C4	178.8 (3)
C11—C10—F1	118.72 (18)	O2—C20—C6	115.10 (19)
C11—C10—C9	123.02 (19)	O2—C20—H20A	108.5
F1—C10—C9	118.24 (19)	C6—C20—H20A	108.5
C10—C11—C12	118.06 (18)	O2—C20—H20B	108.5
C10—C11—H11	121.0	C6—C20—H20B	108.5
C12—C11—H11	121.0	H20A—C20—H20B	107.5
C11—C12—C7	121.10 (18)	O4—C21—O3	123.6 (2)
C11—C12—H12	119.5	O4—C21—C22	123.2 (2)
C7—C12—H12	119.5	O3—C21—C22	113.2 (2)
O1—C13—C7	111.63 (15)	C22^i^—C22—C21	124.2 (3)
O1—C13—C14	103.90 (15)	C22^i^—C22—H22	117.9
C7—C13—C14	109.04 (15)	C21—C22—H22	117.9
			
C6—C1—C2—C3	−1.1 (3)	C6—C1—C13—O1	61.9 (2)
C13—C1—C2—C3	177.22 (18)	C2—C1—C13—C7	120.45 (18)
C1—C2—C3—C4	0.8 (3)	C6—C1—C13—C7	−61.3 (2)
C2—C3—C4—C5	−0.5 (3)	C2—C1—C13—C14	−1.7 (2)
C2—C3—C4—C19	178.5 (2)	C6—C1—C13—C14	176.56 (17)
C3—C4—C5—C6	0.6 (3)	O1—C13—C14—C15	−57.5 (2)
C19—C4—C5—C6	−178.4 (2)	C7—C13—C14—C15	61.7 (2)
C4—C5—C6—C1	−0.9 (3)	C1—C13—C14—C15	−175.25 (17)
C4—C5—C6—C20	178.1 (2)	C13—C14—C15—C16	155.5 (5)
C2—C1—C6—C5	1.2 (3)	C13—C14—C15—C16'	−174.2 (5)
C13—C1—C6—C5	−177.17 (17)	C16'—C15—C16—N1	68.1 (7)
C2—C1—C6—C20	−177.78 (19)	C14—C15—C16—N1	166.0 (5)
C13—C1—C6—C20	3.9 (3)	C17—N1—C16—C15	−86.9 (12)
C12—C7—C8—C9	0.3 (3)	C18'—N1—C16—C15	126.9 (9)
C13—C7—C8—C9	−176.38 (17)	C16'—N1—C16—C15	−71.4 (7)
C7—C8—C9—C10	0.7 (3)	C17'—N1—C16—C15	−114.3 (9)
C8—C9—C10—C11	−1.2 (3)	C18—N1—C16—C15	147.1 (9)
C8—C9—C10—F1	176.93 (17)	C16—C15—C16'—N1	−69.9 (7)
F1—C10—C11—C12	−177.34 (18)	C14—C15—C16'—N1	−163.8 (5)
C9—C10—C11—C12	0.8 (3)	C17—N1—C16'—C15	−127.4 (10)
C10—C11—C12—C7	0.2 (3)	C18'—N1—C16'—C15	86.9 (9)
C8—C7—C12—C11	−0.7 (3)	C16—N1—C16'—C15	66.2 (7)
C13—C7—C12—C11	175.92 (18)	C17'—N1—C16'—C15	−147.9 (8)
C12—C7—C13—O1	13.5 (2)	C18—N1—C16'—C15	113.4 (9)
C8—C7—C13—O1	−169.91 (16)	C3—C4—C19—N2	−164 (14)
C12—C7—C13—C14	−100.7 (2)	C5—C4—C19—N2	15 (14)
C8—C7—C13—C14	75.9 (2)	C5—C6—C20—O2	119.3 (2)
C12—C7—C13—C1	135.18 (18)	C1—C6—C20—O2	−61.7 (3)
C8—C7—C13—C1	−48.3 (2)	O4—C21—C22—C22^i^	−14.6 (4)
C2—C1—C13—O1	−116.40 (18)	O3—C21—C22—C22^i^	164.3 (3)

Symmetry codes: (i) −*x*+2, −*y*+1, −*z*+1.

### Hydrogen-bond geometry (Å, °)

**Table:**

*D*—H···*A*	*D*—H	H···*A*	*D*···*A*	*D*—H···*A*
O1—H1···O2	0.82	1.85	2.655 (2)	165
O2—H2···O3	0.82	1.85	2.642 (3)	162
N1—H1A···O4^ii^	0.91	2.30	3.152 (3)	155
N1—H1A···O3^ii^	0.91	2.04	2.834 (3)	145
C2—H2A···F1^iii^	0.93	2.48	3.392 (3)	167

Symmetry codes: (ii) *x*−1, *y*, *z*; (iii) *x*, *y*−1, *z*.
